# NLRP3 Inflammasome Activation Enhanced by TRIM25 is Targeted by the NS1 Protein of 2009 Pandemic Influenza A Virus

**DOI:** 10.3389/fmicb.2021.778950

**Published:** 2021-11-12

**Authors:** Hong-Su Park, Yao Lu, Kannupriya Pandey, GuanQun Liu, Yan Zhou

**Affiliations:** ^1^Vaccine and Infectious Disease Organization (VIDO), University of Saskatchewan, Saskatoon, SK, Canada; ^2^Vaccinology and Immunotherapeutics Program, School of Public Health, University of Saskatchewan, Saskatoon, SK, Canada

**Keywords:** NLRP3 inflammasome, interleukin-1 beta, caspase-1, tripartite motif-containing protein, TRIM25, influenza A virus, NS1

## Abstract

Nucleotide-binding domain and leucine-rich repeat-containing protein 3 (NLRP3) inflammasome-mediated interleukin-1 beta (IL-1β) production is one of the crucial responses in innate immunity upon infection with viruses including influenza A virus (IAV) and is modulated by both viral and host cellular proteins. Among host proteins involved, we identified tripartite motif-containing protein 25 (TRIM25) as a positive regulator of porcine NLRP3 inflammasome-mediated IL-1β production. TRIM25 achieved this function by enhancing the pro-caspase-1 interaction with apoptosis-associated speck-like protein containing caspase recruitment domain (ASC). The N-terminal RING domain, particularly residues predicted to be critical for the E3 ligase activity of TRIM25, was responsible for this enhancement. However, non-structural protein 1 (NS1) C-terminus of 2009 pandemic IAV interfered with this action by interacting with TRIM25, leading to diminished association between pro-caspase-1 and ASC. These findings demonstrate that TRIM25 promotes the IL-1β signaling, while it is repressed by IAV NS1 protein, revealing additional antagonism of the NS1 against host pro-inflammatory responses.

## Introduction

Interleukin-1 beta (IL-1β) plays a central role in mediating inflammation by recruiting immune cells and supporting the clearance of pathogens. IL-1β is involved in the host immune responses upon infection with viruses including influenza A virus (IAV; Allen et al., 2009; Ichinohe et al., 2009; Thomas et al., 2009). For the regulation of IL-1β production, nucleotide-binding domain and leucine-rich repeat-containing protein 3 (NLRP3) functions as a limiting factor by forming the NLRP3 inflammasome with the adaptor protein, apoptosis-associated speck-like protein containing caspase recruitment domain (ASC), and the effector molecule, pro-caspase-1. Activation of NLRP3 followed by the NLRP3 inflammasome assembly results in the pro-caspase-1 cleavage into active caspase-1, which enables the maturation of pro-IL-1β (Kuriakose and Kanneganti, 2017; Sarvestani and McAuley, 2017).

Viral proteins can either inhibit or activate the NLRP3 inflammasome (Choudhury et al., 2021). On the other hand, host cellular proteins are demonstrated to orchestrate those events. The NLRP3 inflammasome can be positively regulated by spleen tyrosine kinase, NIMA-related kinase 7, protein kinase R, mitochondrial antiviral signaling protein (MAVS; Lu et al., 2012; Subramanian et al., 2013; Lin et al., 2015; He et al., 2016) or negatively regulated by heat shock protein 70 and ephrin type-A receptor 2 (Martine et al., 2019; Zhang et al., 2020). Also, importantly, members of tumor necrosis factor alpha (TNF-α) receptor-associated factors (TRAFs) and tripartite motif-containing proteins (TRIMs) are defined to regulate the NLRP3 inflammasome-mediated IL-1β by functioning as a key enzyme in ubiquitination/SUMOylation. For instance, TRAF3 activates the NLRP3 inflammasome by ASC ubiquitination (Guan et al., 2015; Siu et al., 2019). TRAF6 can either inhibit the NLRP3 inflammasome by ubiquitinating ASC (Chiu et al., 2016), or prime the NLRP3 inflammasome thereby activating the complex (Xing et al., 2017). Among TRIMs, TRIM28, TRIM33, TRIM59, and TRIM62 mediate NLRP3 inflammasome activation with diverse mechanisms (Weng et al., 2014; Liang et al., 2020; Liu and Lei, 2020; Qin et al., 2021). In contrast, negative regulation of the NLRP3 inflammasome by TRIM24, TRIM30, and TRIM31 is demonstrated (Hu et al., 2010; Song et al., 2016; Hang et al., 2021).

Tripartite motif-containing protein 25 (TRIM25), one of the best characterized TRIMs, is involved in host innate immunity including antiviral responses. Its known functions include ubiquitination of an RNA sensor, retinoic acid-inducible gene I (RIG-I), which is essential for upregulating RIG-I-mediated type I interferon (IFN) production. In response to IAV infection, this signaling can be subverted by viral non-structural protein 1 (NS1) that can bind to TRIM25, and amino acids (aa) E96/E97 of NS1 are crucial for the association with TRIM25 (Gack et al., 2009). These residues are shown to be required for IAV NS1 to inhibit the NLRP3 inflammasome activation (Moriyama et al., 2016). Further, TRIM25 deficiency leads to decreased IL-1β production in IAV-infected cells (Pothlichet et al., 2013), suggesting that TRIM25 takes part in the regulation of inflammasome-mediated IL-1β production. However, it is not clear how TRIM25 contributes to the regulation of IL-1β signaling and whether IAV NS1 also targets the IL-1β production modulated by TRIM25.

Our previous investigation showed that porcine IL-1β secretion upon IAV infection was NLRP3 inflammasome-mediated and it was inhibited by NS1 C-terminus of the 2009 human pandemic IAV (Park et al., 2018). In the present study, we set to identify a host cellular protein that enhances the activation of porcine NLRP3 inflammasome. Among others, porcine TRIM25 could upregulate the NLRP3 inflammasome by promoting the interaction between pro-caspase-1 and ASC. This interaction was interfered by NS1 C-terminus of the 2009 pandemic IAV that associates with TRIM25. Thus, this study presented a novel inhibitory function of IAV NS1 on the NLRP3 inflammasome-mediated IL-1β production that is positively regulated by the host cellular TRIM25.

## Materials and Methods

### Cells and Viruses

Human embryonic kidney 293T (HEK293T) cells were cultured using Dulbecco’s modified Eagle’s medium (D5796, Sigma) that was supplemented with 10% fetal bovine serum (FBS; 16000-044, Thermo Fisher) plus 50μg/ml gentamicin (BS724, Bio Basic Canada). Primary porcine alveolar macrophages (PAMs) were isolated from bronchoalveolar lavage fluid of IAV-seronegative piglets and characterized as described (Park et al., 2018) and were cultivated using RPMI 1640 (SH30027.01, GE Healthcare) supplemented with 20% FBS (16000-044, Thermo Fisher), 50μg/ml gentamicin, and 1×Antibiotic-Antimycotic (15240-062, Thermo Fisher). The 2009 human pandemic IAV, influenza A/Halifax/210/2009/H1N1 (Hf09) was propagated in Madin-Darby canine kidney cells. Hf09-816 and Hf09-817, two isogenic viruses with truncated NS1 (C-terminal aa 74–219 deleted for Hf09-816 and aa 100–219 deleted for Hf09-817), were generated and propagated as described in our earlier study (Park et al., 2018).

### Plasmid Construction

Expression plasmids for porcine NLRP3, ASC, pro-caspase-1, and pro-IL-1β were generated using cDNA from primary PAMs as previously described (Park et al., 2018). Using the pro-caspase-1-expressing construct, the full-length pro-caspase-1 and its N-terminal caspase activation and recruitment domain (CARD; aa 1–91) were cloned into pCMV-3×Flag (N-terminal tag) for generating Flag-pro-caspase-1 and Flag-CARD. The full-length porcine TRIM25, TRAF3, TRAF6, and MAVS were amplified using the cDNA from primary PAMs or a porcine macrophage cell line, 3D4/2 for MAVS. Each of them was cloned into pcDNA3.1-3×Myc (C-terminal tag) to generate TRIM25-Myc, TRAF3-Myc, TRAF6-Myc, and MAVS-Myc, respectively. Porcine TRIM25 was also cloned into pcDNA3.1-3×HA (N-terminal tag) to generate HA-TRIM25, which was further used as the template to generate the following mutants: a mutant TRIM25 construct with C13A/C16A mutation [HA-TRIM25 (C13A/C16A)]; a truncated TRIM25 construct with the N-terminal RING domain (aa 1–54) deletion [HA-TRIM25 (delRING)]. Hf09 NS1_100-219_-Myc, which is NS1 C-terminal aa 100–219 of the IAV Hf09 strain cloned into pcDNA3.1-3×Myc (C-terminal tag), was constructed in our previous study (Park et al., 2018). Primers were designed based on the GenBank sequences of porcine pro-caspase-1, TRIM25, TRAF3, TRAF6, and MAVS [accession numbers: AK231984 (pro-caspase-1), XM_005656971 (TRIM25), XM_021081623 (TRAF3), EU095967 (TRAF6), and AB287431 (MAVS)].

### NLRP3 Inflammasome Reconstitution Assay

Human embryonic kidney 293T cells were seeded at 1.5×10^5^ cells per well on 24-well plates and cultured overnight. Each well was co-transfected with plasmids expressing porcine NLRP3 inflammasome components and pro-IL-1β [NLRP3 (30ng), ASC (20ng), pro-caspase-1 (20ng), and pro-IL-1β (100ng)] along with other plasmids (300ng of Myc-vector or Myc-tagged porcine TRIM25, TRAF3, or TRAF6, 300ng of HA-vector or HA-tagged porcine TRIM25 WT/mutants, or indicated amounts of TRIM25-Myc) using *Trans*IT-LT1 Transfection Reagent (MIR2300, Mirus Bio). At 16 or 24h post-transfection (hpt), supernatants were harvested for porcine IL-1β ELISA and the cells were lysed with 1×sodium dodecyl sulfate (SDS) sample buffer containing 1% β-mercaptoethanol to be analyzed by Western blotting.

### Porcine IL-1β ELISA

Clear round-bottom 96-well plates (3655, Thermo Fisher) were coated with 2μg/ml of mouse anti-porcine IL-1β antibody (MAB6811, R&D Systems) in phosphate-buffered saline (PBS) overnight. Wells were blocked with 1% bovine serum albumin (BSA; A7030, Sigma) in PBS for 1h at room temperature. Either test samples or the standard were added into wells followed by 2h incubation. Two-fold serial dilutions of the recombinant porcine IL-1β (681-PI-010, R&D Systems) were prepared in diluent [0.1% BSA in Tris-buffered saline (TBS) with 0.05% Tween 20] to generate the standard curves. The plates were incubated for 1h with 50ng/ml of goat anti-porcine IL-1β biotinylated antibody (BAF681, R&D Systems) prepared in the diluent, and for another 1h with alkaline phosphatase-conjugated streptavidin (016-050-084, Jackson ImmunoResearch) that was 5,000-fold diluted in the diluent. The plates were further incubated after adding 1mg/ml of p-nitrophenyl phosphate in diethanolamine buffer (1M diethanolamine, 0.5M MgCl_2_, pH 9.8), and optical densities were measured at 405nm with reference at 490nm by using an xMark Microplate Absorbance Spectrophotometer (Bio-Rad).

### Co-immunoprecipitation

Human embryonic kidney 293T cells were seeded at 9×10^5^ per well on 6-well plates and were transfected with 1μg each of an empty vector and/or a plasmid expressing an epitope-tagged target protein as indicated. At 24hpt, the cells were lysed with 500μl of cell lysis buffer (50mM Tris, pH 7.4, 150mM NaCl, 0.5% Nonidet P-40 substitute, and 1×protease inhibitor cocktail) on ice, and the lysates were clarified by centrifugation at 12,000×g at 4°C for 10min. For input samples, 10% of the clarified lysates were mixed with 5×SDS sample buffer containing 5% β-mercaptoethanol and boiled at 95°C for 5min. Dynabeads Protein G (10004D, Thermo Fisher; 35μl per each lysate) was conjugated with 1μg of either mouse anti-FLAG antibody (F3165, Sigma), mouse anti-HA antibody (ab9110, Abcam), or mouse IgG1 isotype control antibody (5415, Cell Signaling Technology) in 300μl PBS containing 0.02% Tween 20 by agitation at room temperature for 1h. The beads were incubated with the cell lysates at room temperature for 2h while being agitated. After washed with TBS, the beads were mixed with 60μl of 2×SDS sample buffer containing 2% β-mercaptoethanol and boiled as above. The eluted proteins and input samples were analyzed by SDS-polyacrylamide gel electrophoresis (SDS-PAGE) and Western blotting.

### Western Blotting and Antibodies

Whole cell lysates or immunoprecipitation (IP) samples were subjected to SDS-PAGE. The resolved proteins were blotted to 0.45μm nitrocellulose membranes (1620115, Bio-Rad), which were further blocked with 5% skim milk in Tris-buffered saline with 0.1% Tween 20 (TBST) for 1h. The membranes were incubated with primary antibodies in TBST at 4°C overnight and with secondary antibodies in TBST at room temperature for 1h. An Odyssey Infrared Imager (LI-COR Biosciences) was used to scan the membranes.

The following primary antibodies were used: goat anti-porcine IL-1β antibody (BAF681, R&D Systems); rabbit anti-porcine caspase-1 (p20) antibody (PAB592Po01, Cloud-Clone Corp.); rabbit anti-DYKDDDDK tag antibody (2368, Cell Signaling Technology); rabbit anti-Myc-tag antibody (2278, Cell Signaling Technology); mouse anti-Myc-tag antibody (2276, Cell Signaling Technology); mouse anti-β-actin antibody (3700, Cell Signaling Technology); mouse anti-FLAG antibody (F3165, Sigma); mouse anti-HA antibody (ab9110, Abcam); mouse anti-TRIM25 antibody (sc-271254, Santa Cruz); and rabbit anti-NP antibody that was previously produced in our lab (Shin et al., 2007). Secondary antibodies from LI-COR Biosciences were used as follows: IRDye 680RD donkey anti-rabbit (926-68073); IRDye 800CW donkey anti-mouse (926-32212); and IRDye 800CW donkey anti-goat (926-32214) antibodies.

Band intensities were quantified by using ImageJ (National Institutes of Health, United States). Relative intensities of proteins in the whole cell lysates were shown after normalization to intensities of β-actin. Relative intensities of interacting proteins were shown after normalization to intensities of immunoprecipitated target proteins for the IP samples.

### Quantitative PCR

Total RNA was extracted from HEK293T cells transfected as indicated by using RNeasy Mini kit (74104, Qiagen). After treatment with DNase I (18068-015, Thermo Fisher), RNA was subjected to the first-strand cDNA synthesis by using SuperScript III Reverse Transcriptase (18080044, Thermo Fisher). Real-time PCR was conducted with each cDNA mixed with 0.5μM each of forward and backward primers, and Power SYBR Green PCR Master Mix (4367659, Thermo Fisher) under a standard protocol.

Porcine pro-caspase-1 expression was assessed by using forward (5'-GGTACGATCAATGGCCTCTT-3') and reverse (5'-TCGGGCCTTATCCATAACTG-3') primers designed based on the GenBank sequence (accession number AK231984). Human glyceraldehyde-3-phosphate dehydrogenase (GAPDH) was selected as the housekeeping gene and was amplified with forward (5'-TGCACCACCAACTGCTTAGC-3') and reverse (5'-GGCATGGACTGTGGTCATGAG-3') primers designed based on the GenBank sequence (accession number NM_002046). Based on the threshold cycle (Ct) of the target gene and GAPDH, 2^-(ΔΔCt)^ calculation was used to measure the relative expression of the target gene by normalizing to the expression level of GAPDH.

### Statistical Analysis

One-way ANOVA with Tukey’s multiple comparisons tests was performed using GraphPad Prism 7. The error bars represent the mean±SD. The *p* values of less than 0.05 were considered statistically significant.

## Results

### TRIM25 Promotes the NLRP3 Inflammasome Activity

**Figure 1:**
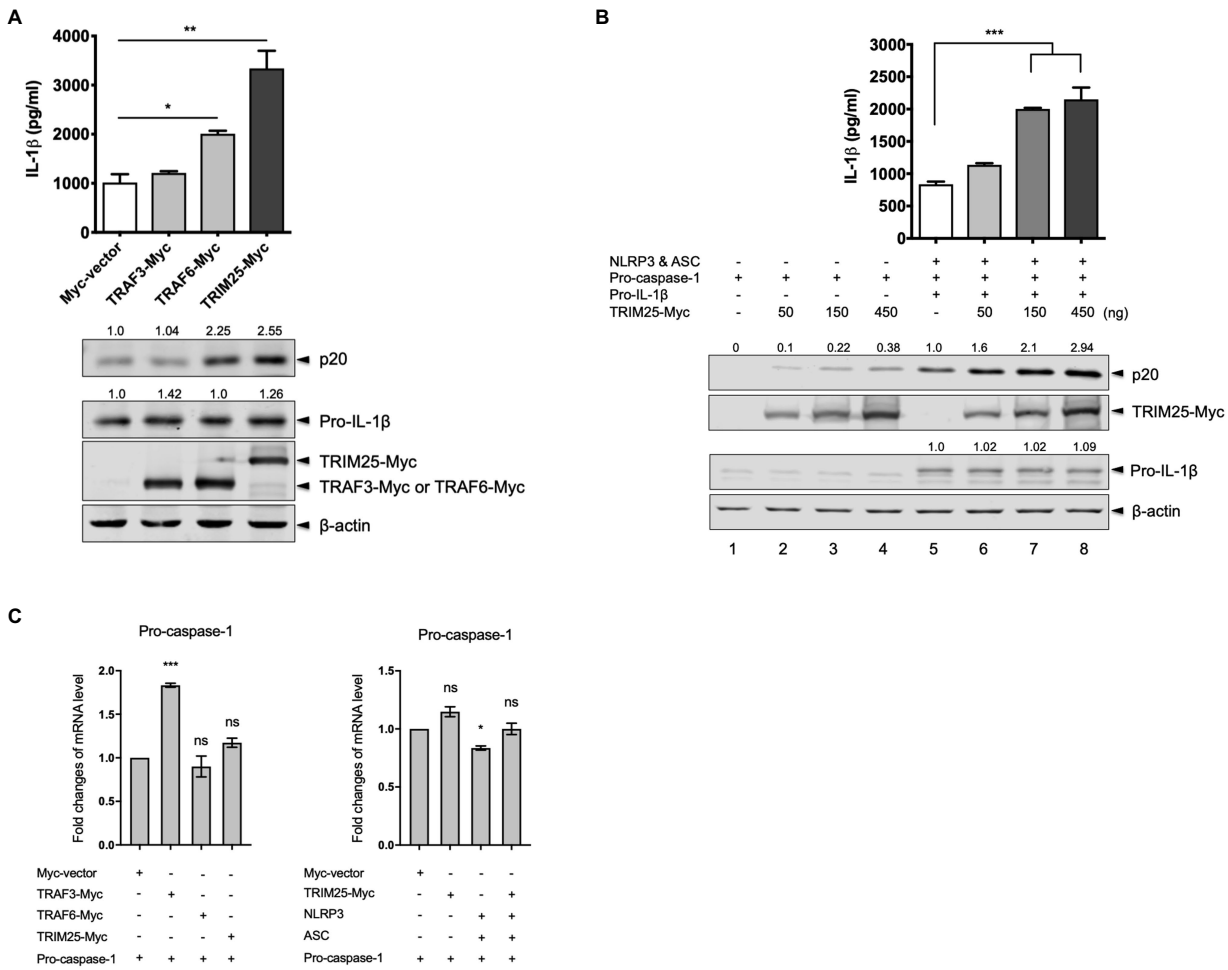
Tripartite motif-containing protein 25 (TRIM25) enhances the nucleotide-binding domain and leucine-rich repeat-containing protein 3 (NLRP3) inflammasome activity. **(A)** Human embryonic kidney 293T (HEK293T) cells were transfected with plasmids expressing porcine NLRP3, apoptosis-associated speck-like protein containing caspase recruitment domain (ASC), pro-caspase-1, and pro-interleukin-1 beta (IL-1β) along with an empty Myc-vector or a plasmid expressing Myc-tagged porcine TRAF3, TRAF6, or TRIM25 for 16h. Porcine IL-1β levels from cell-free supernatants were measured by ELISA. From cell lysates, the expression of proteins (p20 subunit of active caspase-1, pro-IL-1β, Myc-tagged TRAF3, TRAF6, and TRIM25) was analyzed by Western blotting, while the endogenous β-actin level was monitored as a loading control. Relative intensities of p20 and pro-IL-1β to that in the sample with Myc-vector were shown above each band. **(B)** HEK293T cells were transfected with a plasmid expressing porcine pro-caspase-1 (lanes 1–4) or plasmids expressing porcine NLRP3 inflammasome components plus pro-IL-1β (lanes 5–8) along with increasing amounts of Myc-tagged porcine TRIM25. An empty Myc-vector was included where needed to keep the total DNA amount constant. Porcine IL-1β secretion and protein expression as indicated at 16h were analyzed as in **(A)**. Relative intensities of p20 (lanes 1–8) and pro-IL-1β (lanes 5–8) to that in lane 5 were shown as in **(A)**. **(C)** HEK293T cells were transfected with plasmids as indicated for 18h. Pro-caspase-1 mRNA levels were measured by quantitative PCR and expressed as fold changes over the expression level in cells transfected with pro-caspase-1 construct and an empty Myc-vector. Statistical analysis was performed with one-way ANOVA. ns, not significant; ^*^p<0.05; ^**^p<0.01; and ^***^p<0.001. Results are representative of three independent experiments.

TRIM25 is involved in the regulation of innate immunity, while its role in IL-1β signaling is elusive. TRAF3 and TRAF6 are reported to regulate the NLRP3 inflammasome activation. We were interested in their effects on porcine NLRP3 inflammasome-mediated IL-1β production. Using cDNA from total RNA of primary PAMs, porcine TRIM25, TRAF3, and TRAF6 were cloned into a Myc-tagged expression vector. To check how the ectopically expressed proteins contribute to the NLRP3 inflammasome regulation, we reconstituted the NLRP3 inflammasome in HEK293T cells that are deficient in human NLRP3 inflammasome (Bryan et al., 2009). The cells were co-transfected with plasmids expressing porcine NLRP3, ASC, pro-caspase-1, and pro-IL-1β along with a plasmid expressing TRAF3, TRAF6, or TRIM25. Compared to the cells transfected with a Myc-vector or Myc-tagged TRAF3, the expression of an active caspase-1 subunit, p20 and the secretion of porcine IL-1β in the supernatant were significantly increased in the cells transfected with the TRIM25 construct (Figure 1A). Pro-IL-1β expression levels were not significantly changed among these conditions. Increased p20 expression and IL-1β secretion in TRAF6-transfected cells were also observed as described by a report that TRAF6 positively regulates the NLRP3 inflammasome activity in mice (Xing et al., 2017). Since the upregulation of IL-1β production by TRIM25 was more significant than that by TRAF6 and this dramatic effect on IL-1β signaling by TRIM25 has not been reported, we focused on the roles of TRIM25 on NLRP3 inflammasome activation. To further confirm that TRIM25 plays a positive role on NLRP3 inflammasome-mediated IL-1β production, NLRP3 inflammasome reconstitution was performed in another setting where increasing amounts of the TRIM25-expressing plasmid were included in each condition. In addition, cells were transfected with the increasing amounts of the TRIM25 construct plus a plasmid expressing pro-caspase-1, but not NLRP3, ASC and pro-IL-1β to check how TRIM25 overexpression affects pro-caspase-1 autocleavage without other NLRP3 inflammasome components. TRIM25 overexpression induced a dose-dependent increase of porcine pro-caspase-1 cleavage as indicated by p20 expression (Figure 1B, lanes 1–4). In the condition where the NLRP3 inflammasome components plus pro-IL-1β were expressed, p20 expression was more obviously increased by TRIM25 expression and it was also TRIM25 dose-dependent (Figure 1B, lanes 5–8). Concomitantly, IL-1β secretion in the supernatants detected by ELISA was TRIM25 dose-dependent for these conditions, whereas pro-IL-1β expression was not affected. This showed that a positive contribution of TRIM25 to IL-1β production was mediated by increased pro-caspase-1 cleavage, and this effect was more evident when NLRP3 and ASC were present. To examine whether the increased caspase-1 activity by TRIM25 is due to the transcriptional increase, mRNA expression of pro-caspase-1 was measured by quantitative PCR after HEK293T cells were transfected with a construct expressing pro-caspase-1 and those expressing other proteins. Pro-caspase-1 transcription levels were not significantly changed by overexpression of TRIM25 or TRAF6, while a moderate increase was shown with TRAF3, when normalized to the condition with an empty vector (Figure 1C, left). With NLRP3 and ASC overexpressed, the effect of TRIM25 on the pro-caspase-1 transcription was not significant either (Figure 1C, right).

### TRIM25 Enhances the Interaction Between pro-caspase-1 and ASC

**Figure 2:**
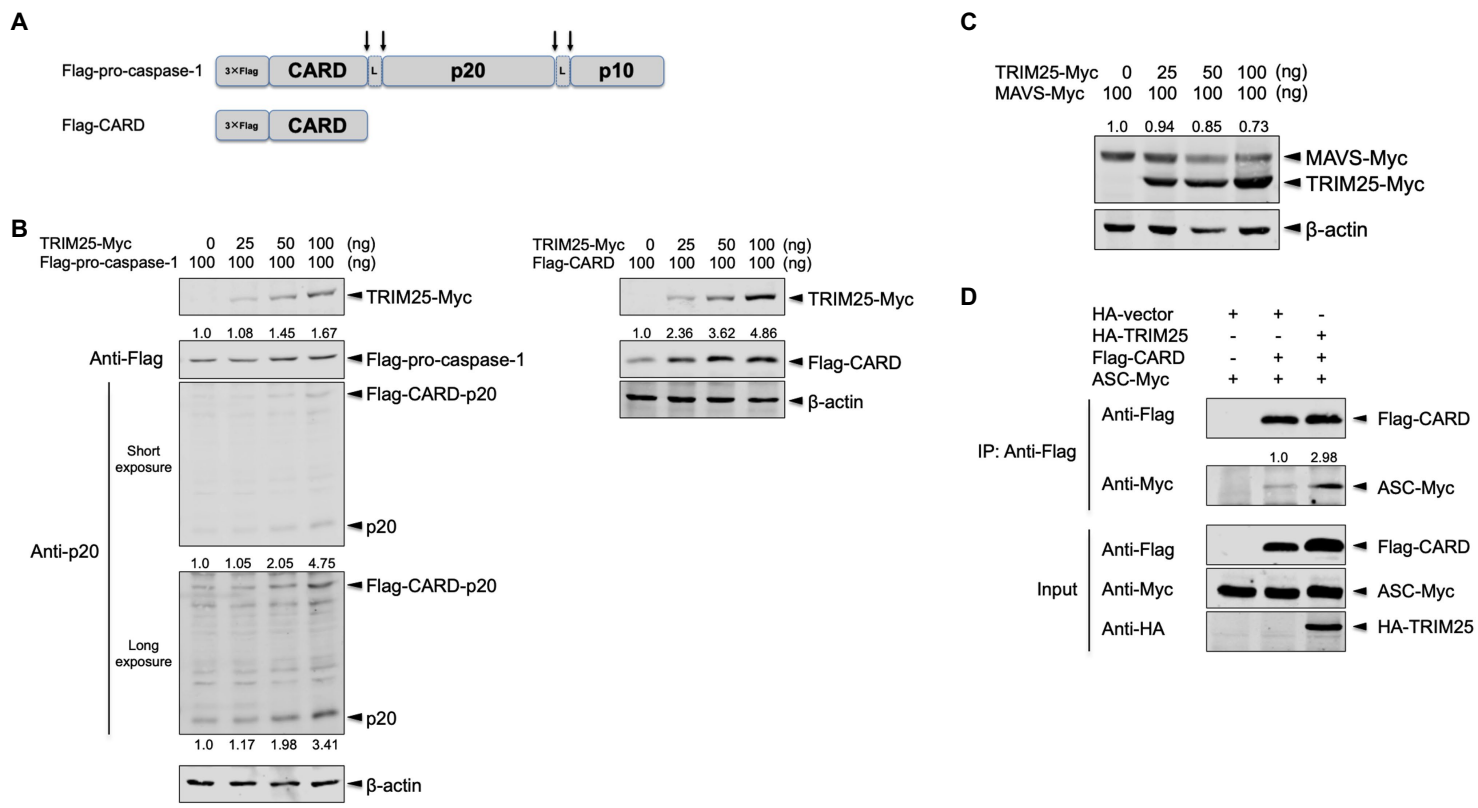
TRIM25 promotes the interaction between pro-caspase-1 caspase activation and recruitment domain (CARD) and ASC. **(A)** Domain architectures of 3× Flag-tagged full-length porcine pro-caspase-1 expressed by the Flag-pro-caspase-1 construct and 3× Flag-tagged porcine pro-caspase-1 CARD expressed by the Flag-CARD construct. Pro-caspase-1 CARD interacts with ASC for inflammasome activation. Upon cleavage on the sites indicated by arrows, active subunits (p20 and p10) are generated. The linker regions are denoted by L. **(B)** HEK293T cells were transfected with a plasmid expressing Flag-tagged porcine pro-caspase-1 (left panel) or Flag-tagged porcine pro-caspase-1 CARD (right panel) along with increasing amounts of a plasmid expressing Myc-tagged porcine TRIM25 for 16h. The expression of Myc-tagged TRIM25, Flag-tagged pro-caspase-1, Flag-tagged CARD-p20 fragment, p20 and Flag-tagged CARD was analyzed by Western blotting. Relative intensities of Flag-pro-caspase-1 (left panel) or Flag-CARD (right panel) to that without TRIM25-Myc were shown above each band. Relative intensities of Flag-CARD-p20 and p20 were shown above and below each band, respectively, on the long-exposure image (left panel). **(C)** HEK293T cells were transfected with a plasmid expressing Myc-tagged porcine mitochondrial antiviral signaling protein (MAVS) along with increasing amounts of a plasmid expressing Myc-tagged porcine TRIM25 for 16h. The expression of Myc-tagged MAVS and TRIM25 was analyzed by Western blotting. Relative intensities of MAVS-Myc to that in sample without TRIM25-Myc were shown above each band. **(D)** HEK293T cells were transfected with plasmids expressing Flag-tagged porcine pro-caspase-1 CARD and Myc-tagged porcine ASC along with either an empty HA-vector or a plasmid expressing HA-tagged porcine TRIM25 for 24h. Cell lysates were subjected to IP with anti-Flag antibody. The expression of Flag-tagged CARD, Myc-tagged ASC and HA-tagged TRIM25 in IP and input samples was analyzed by Western blotting using antibodies indicated. A relative intensity of ASC-Myc to that in sample without HA-TRIM25 was shown above each band. Results are representative of three independent experiments.

Pro-caspase-1 is consisted of N-terminal CARD and two active subunits, p20 and p10 that are generated through autocleavage (Figure 2A). While two of each active subunit compose a tetramer of p20/p10 to process pro-IL-1β molecules, CARD-p20 and p10 subunits are reported to be the dominant form before p20/p10 is generated (Boucher et al., 2018). To further dissect the increased caspase-1 activation by TRIM25, the full-length pro-caspase-1 and its active subunit, p20 were assessed together by expressing Flag-tagged pro-caspase-1 plus a low amount of Myc-tagged TRIM25. The cells were transfected with a plasmid expressing the full-length porcine pro-caspase-1 tagged with 3×Flag in its N-terminus (namely, Flag-pro-caspase-1) as depicted in the Figure 2A along with the increasing amounts of the TRIM25 construct. TRIM25 moderately augmented the expression of the full-length pro-caspase-1 and cleavage of it, and as a result, there was more expression of the CARD-p20 fragment and the p20 subunit, which was more obviously shown with a long-exposure image (Figure 2B, left). These data reaffirmed that TRIM25 can increase caspase-1 activity.

Since TRIM25 modifies the N-terminal domain CARD of RIG-I (Gack et al., 2007), we hypothesized that pro-caspase-1 CARD is the main domain regulated by TRIM25. To explore the TRIM25’s effect on the pro-caspase-1 CARD, Flag-pro-caspase-1 CARD (namely, Flag-CARD) was constructed (Figure 2A). When the cells were transfected with a plasmid expressing Flag-CARD along with the increasing amounts of the TRIM25 construct, the expression level of Flag-CARD was TRIM25 dose-dependent (Figure 2B, right) and this enhancement was more prominent than what is observed for the full-length pro-caspase-1. This indicates that TRIM25-mediated upregulation of caspase-1 activity is mainly derived from its action on the CARD of pro-caspase-1. To exclude a possibility that the observed TRIM25 effect is non-specific, porcine MAVS was expressed with TRIM25. MAVS also harbors CARD in its N-terminus and is degraded by TRIM25 (Castanier et al., 2012). Indeed, the expression of porcine MAVS was reduced when expressed with TRIM25 (Figure 2C).

We questioned how the interaction between pro-caspase-1 CARD and ASC is regulated by TRIM25, since their association is prerequisite for inflammasome activation (Kesavardhana and Kanneganti, 2017). Overexpression of the full-length caspase-1 induces cell death (Denes et al., 2012), especially in a setting that require a substantial amount of expressed proteins such as co-immunoprecipitation (Co-IP). Therefore, we used the Flag-CARD construct to examine the interaction between pro-caspase-1 and ASC. Flag-CARD and Myc-tagged full-length ASC (namely, ASC-Myc) were expressed along with HA-tagged TRIM25, and the cell lysates were subjected to Co-IP. The CARD expression was elevated by porcine TRIM25 expression compared to the condition with HA-vector, and the interaction of the CARD with ASC was also increased as a result (Figure 2D). This demonstrates that TRIM25-mediated upregulation of the NLRP3 inflammasome activity is achieved by enhanced interaction between pro-caspase-1 and ASC.

### TRIM25 RING Domain Is Responsible for the NLRP3 Inflammasome Activation

**Figure 3:**
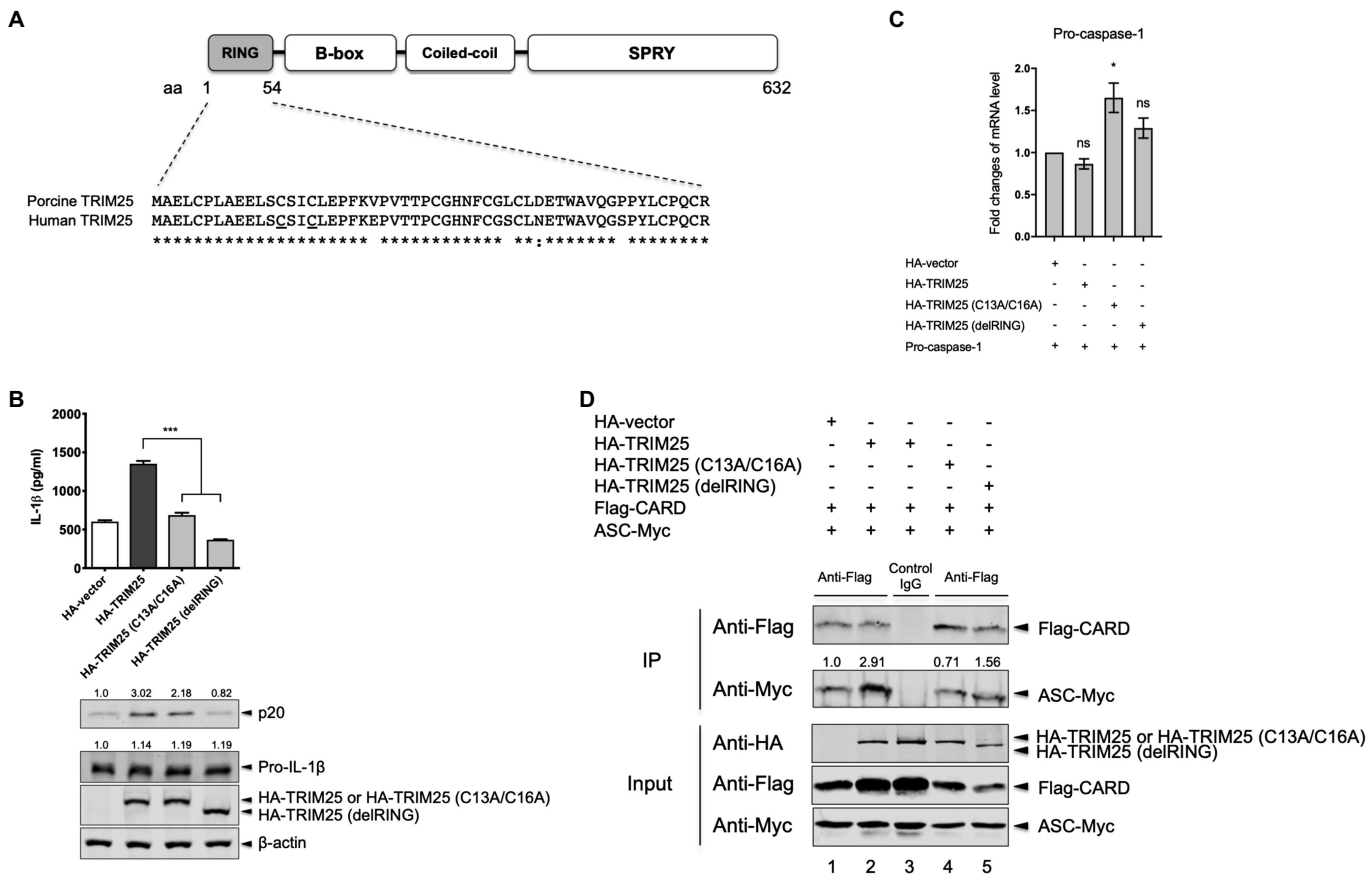
TRIM25 RING domain is responsible for promoting the NLRP3 inflammasome activation. **(A)** Domain architecture of TRIM25 and amino acid sequence alignment of the RING domain between porcine and human TRIM25. The sequences are based on the GenBank accession numbers XM_005656971 for porcine TRIM25 and NM_005082 for human TRIM25. Amino acid positions are based on the sequence of porcine TRIM25. Residues (C13 and C16) known to be critical for the E3 ligase activity of human TRIM25 are underlined. Consensus symbols indicate as follows: asterisk (^*^), fully conserved residues; colon (:), residues that share strongly similar properties. **(B)** HEK293T cells were transfected with plasmids expressing porcine NLRP3 inflammasome components and pro-IL-1β along with an empty HA-vector or a plasmid expressing HA-tagged porcine TRIM25 (WT or mutants) for 16h. Porcine IL-1β levels from cell-free supernatants were measured by ELISA. From cell lysates, the expression of proteins (p20, pro-IL-1β, and HA-tagged TRIM25) was analyzed by Western blotting. Relative intensities of p20 and pro-IL-1β to that in the samples with HA-vector were shown above each band. **(C)** HEK293T cells were transfected with plasmids as indicated for 18h. Pro-caspase-1 mRNA levels were measured by quantitative PCR and expressed as fold changes over the expression level in cells transfected with pro-caspase-1 construct and an empty HA-vector. **(D)** HEK293T cells were transfected with plasmids expressing Flag-tagged porcine pro-caspase-1 CARD and Myc-tagged porcine ASC along with either an empty HA-vector or plasmids expressing TRIM25 (WT or mutants) for 24h. Cell lysates were subjected to IP with mouse IgG isotype control or anti-Flag antibody. The expression of Flag-tagged CARD, Myc-tagged ASC, and HA-tagged TRIM25 (WT or mutants) in IP and input samples was analyzed by Western blotting using antibodies indicated. Relative intensities of ASC-Myc to that in lane 1 were shown above each band. Statistical analysis was performed with one-way ANOVA. ns, not significant; ^*^p<0.05; ^***^p<0.001. Results are representative of three independent experiments.

We sought to identify the region or residues in TRIM25 that contributes to the NLRP3 inflammasome activation. The RING domain of TRIM25 is critical for the E3 ligase activity (Gack et al., 2007). For human TRIM25, C13 and C16 in the RING domain are known to be required for its E3 ligase activity (Meyerson et al., 2017) and these two residues are conserved between porcine and human TRIM25 (Figure 3A). Thus, we focused on testing deletion of the RING domain or ablation of the potential E3 ligase activity in porcine TRIM25. To be used in NLRP3 inflammasome reconstitution assay, mutant porcine TRIM25 constructs were generated using HA-tagged WT TRIM25 (namely, HA-TRIM25): C13 and C16 were each replaced by alanine in HA-TRIM25 (C13A/C16A); and RING domain (aa 1–54) was deleted in HA-TRIM25 (delRING). Compared to WT TRIM25, caspase-1 p20 expression was significantly reduced when the RING domain was deleted, and it was moderately reduced when C13 and C16 within the RING domain were mutated (Figure 3B). In parallel, IL-1β levels were also lower in the supernatant of cells overexpressing the mutant TRIM25. Additionally, the effects of WT or two mutants TRIM25 on the pro-caspase-1 transcription were evaluated by quantitative PCR (Figure 3C). Pro-caspase-1 transcription levels were not decreased by TRIM25 mutants, either TRIM25 (C13A/C16A) or TRIM25 (delRING) compared to WT TRIM25.

To further assess how the interaction of pro-caspase-1 CARD with ASC is influenced by WT/mutant TRIM25, Co-IP was performed after Flag-CARD and ASC-Myc were expressed along with HA-tagged TRIM25 (WT, C13A/C16A or delRING version). As observed above, the CARD expression was increased by the expression of WT TRIM25, compared to the condition with HA-vector. However, it was not increased by the mutant TRIM25, either C13A/C16A or delRING (Figure 3D). Consequently, the amount of ASC-Myc co-immunoprecipitated with Flag-CARD was decreased in the case of mutant TRIM25 compared to the condition with WT TRIM25. The results indicate that porcine TRIM25’s RING domain, more specifically, the residues C13 and C16 that are postulated to be required for the E3 ligase activity, is critical for TRIM25 to promote the NLRP3 inflammasome activity.

### NS1 C-Terminus of 2009 Pandemic IAV Interacts With TRIM25 and Suppresses the Interaction Between pro-caspase-1 and ASC

**Figure 4:**
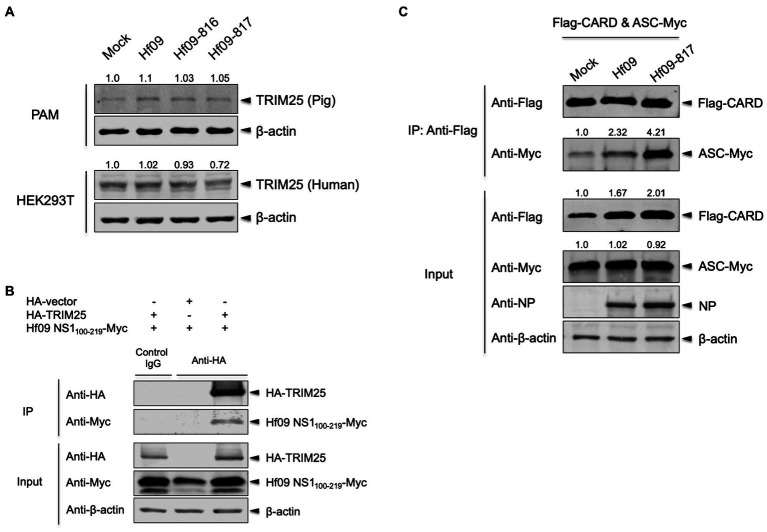
NS1 C-terminus of 2009 pandemic IAV interacts with TRIM25 and inhibits the interaction between pro-caspase-1 and ASC. **(A)** PAMs or HEK293T cells were either mock-infected or infected with Hf09, Hf09-816, or Hf09-817 at an MOI of 5 for 16h. The expression of endogenous porcine or human TRIM25 was analyzed by Western blotting. Relative intensities of TRIM25 to the mock condition were shown above each band. **(B)** HEK293T cells were transfected with an empty HA-vector or a plasmid expressing HA-tagged porcine TRIM25 along with a plasmid expressing Myc-tagged Hf09 NS1 C-terminus (aa 100–219) for 24h. Cell lysates were subjected to IP with mouse IgG isotype control or anti-HA antibody. The expression of HA-tagged TRIM25 and Myc-tagged NS1 C-terminus in IP and input samples was analyzed by Western blotting using antibodies indicated. **(C)** HEK293T cells were transfected with plasmids expressing Flag-tagged porcine pro-caspase-1 CARD and Myc-tagged porcine ASC for 16h, and then either mock-infected or infected with Hf09 or Hf09-817 at an MOI of 10 for 8h. Cell lysates were subjected to IP with anti-Flag antibody. The expression of Flag-tagged CARD, Myc-tagged ASC, and viral NP in IP and input samples was analyzed by Western blotting using antibodies indicated. Relative intensities of ASC-Myc to the mock condition were shown above each band. Results are representative of three independent experiments.

**Figure 5:**
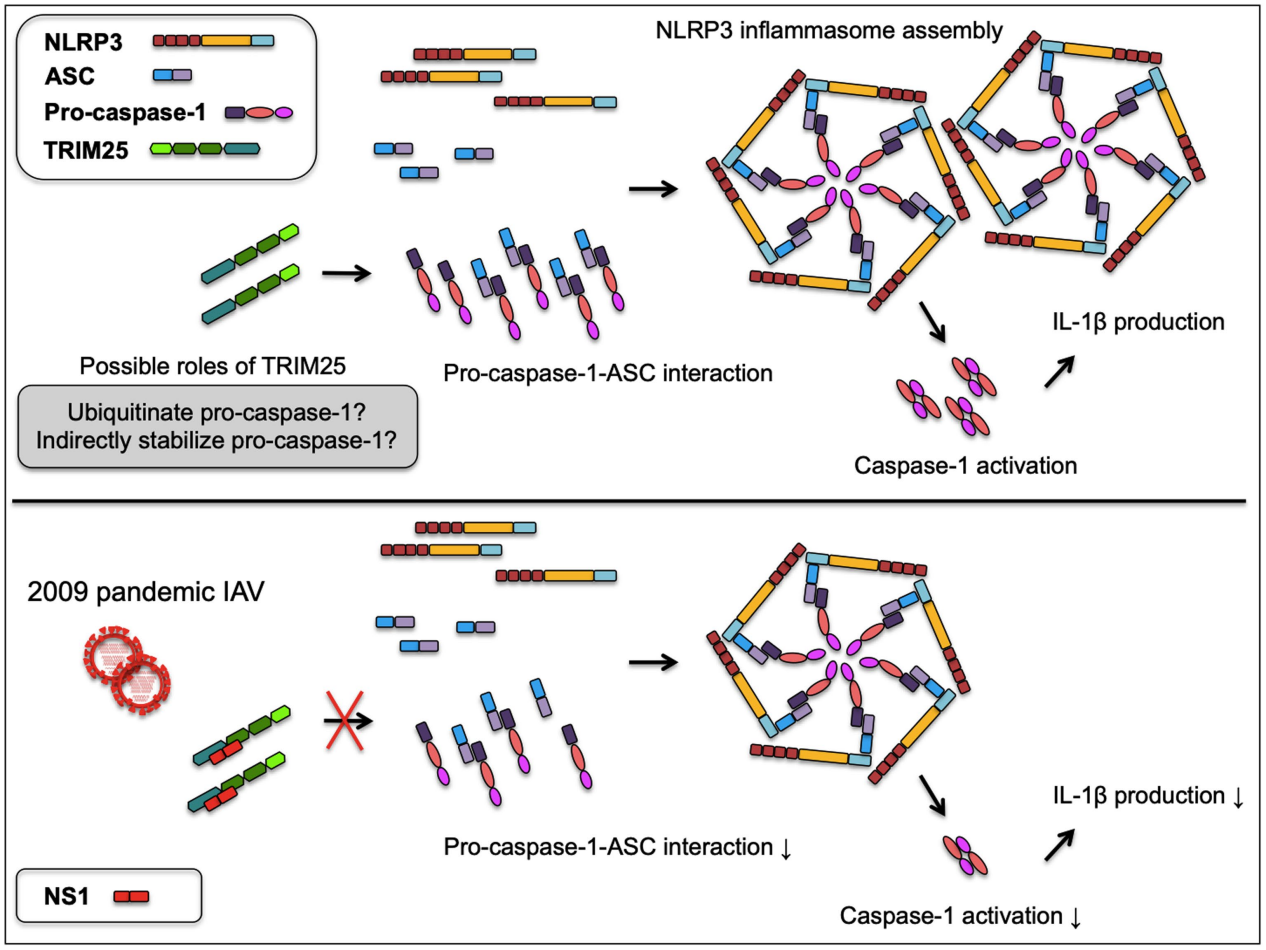
A proposed model of NS1 targeted TRIM25-mediated NLRP3 inflammasome activation. TRIM25 promotes the interaction between pro-caspase-1 and ASC by acting on the N-terminal CARD of pro-caspase-1. TRIM25 may possibly ubiquitinate the pro-caspase-1 CARD or indirectly contribute to the stabilization of pro-caspase-1, while the mechanisms are yet to be determined. Increased pro-caspase-1-ASC interaction leads to the enhanced assembly of NLRP3 inflammasome and caspase-1 activation required for IL-1β production. Upon infection with 2009 pandemic IAV, the NS1 C-terminus binds TRIM25 to disrupt the pro-caspase-1-ASC interaction, resulting in the decreased caspase-1 activity and IL-1β production.

IAV NS1 is a well-known inhibitor of the host inflammatory and antiviral responses. NS1 C-terminus was shown to inhibit the NLRP3 inflammasome-mediated IL-1β production in our previous study using the 2009 human pandemic H1N1 strain, Hf09 and its isogenic viruses with NS1 C-terminal deletion, Hf09-816 (aa 74–219 deleted) and Hf09-817 (aa 100–219 deleted; Park et al., 2018). To investigate if NS1 C-terminus can target the TRIM25-mediated NLRP3 inflammasome activation, we first examined whether NS1 C-terminus inhibits the expression of TRIM25. PAMs or HEK293T cells were infected with Hf09 or two isogenic viruses (Hf09-816 or Hf09-817) whose NS1 C-termini were deleted. The expression of endogenous TRIM25 in PAMs was not significantly changed upon infection with Hf09 and the two viruses harboring the NS1 C-terminal deletion (Figure 4A, upper). In HEK293T cells, the expression of endogenous human TRIM25 was not elevated by infection with the two NS1-mutant viruses either (Figure 4A, lower). Next, we explored if NS1 C-terminus interacts with TRIM25. HA-tagged TRIM25 and Myc-tagged Hf09 NS1 C-terminus (aa 100–219) were co-expressed in HEK293T cells and Co-IP was performed. Immunoprecipitation of Hf09 NS1_100-219_ with porcine TRIM25 was confirmed when compared to the condition using an isotype control antibody (Figure 4B). In an effort to observe whether NS1 C-terminus is responsible for inhibiting TRIM25-mediated NLRP3 inflammasome activation, we also checked if the interaction between pro-caspase-1 and ASC is differently regulated by WT Hf09 (with NS1 aa 1–219) and one of its mutant viruses, Hf09-817 (with NS1 aa 100–219 deleted). Co-IP was performed after HEK293T cells were transfected with plasmids expressing Flag-CARD and ASC-Myc, and then, was infected with either Hf09 or Hf09-817. Upon infection with Hf09 or Hf09-817, the expression of Flag-CARD was increased compared to mock infection, whereas ASC-Myc expression was hardly affected (Figure 4C). However, the co-immunoprecipitation of ASC-Myc with Flag-CARD was significantly increased in the cells infected with Hf09-817 in comparison to those infected with Hf09. These show that NS1 C-terminus, by interacting with TRIM25, targets the function of TRIM25 that enhances the interaction of pro-caspase-1 CARD with ASC as shown in the proposed model (Figure 5).

## Discussion

NLRP3 inflammasome-mediated IL-1β production is fine-tuned, but in multifaceted ways. In response to viral infection, it is controlled by viral proteins as well as host cellular proteins that upregulate or downregulate the process. TRIM family member proteins have recently attracted considerable attention due to multiple roles on the NLRP3 inflammasome. There is a variation in the number of TRIM genes expressed by different species. TRIM25 is one of several TRIM members that are activated in both human and pig cells in response to IFN treatment, and the transcription of porcine TRIM25 is increased in PAMs infected with RNA viruses of concern (Wei et al., 2019), indicating porcine TRIM25 as an important host factor.

We first identified porcine TRIM25’s roles on the NLRP3 inflammasome-mediated IL-1β production by utilizing the previously set-up porcine NLRP3 inflammasome reconstitution system (Park et al., 2018). TRIM25 promoted pro-caspase-1 cleavage without significantly affecting the transcriptional level of pro-caspase-1. Also, TRIM25 did not influence the expression of pro-IL-1β, which is induced by nuclear factor kappa B (NF-κB) signaling (Kesavardhana and Kanneganti, 2017). It does not seem to be NF-κB-mediated increase of NLRP3 inflammasome activity by TRIM25 occurring in our setting without any type of agonist involved. Rather, TRIM25 may directly or indirectly stabilize the pro-caspase-1. In comparison, RIG-I-mediated NF-κB activation can be increased by TRIM25 (Gack et al., 2007); in case NF-κB activation is mediated by TNF-α treatment, the NF-κB activity shown by, for example, increased transcription of pro-IL-1β, is further upregulated by TRIM25 (Liu et al., 2020). On the contrary, when pro-caspase-1 cleavage and IL-1β production upon NLRP1 inflammasome activation are induced by doxorubicin, those events can be downregulated by TRIM25 (Wang et al., 2021), implying that TRIM25 exerts two-sided roles depending on which signaling it is involved.

We showed that while the enhanced cleavage of pro-caspase-1 was observed when it was solely expressed with TRIM25, the effect of TRIM25 on caspase-1 activation was more remarkable in the presence of NLRP3 and ASC. This may suggest that the formation of NLRP3 inflammasome complex can be more solid or firmly maintained when pro-caspase-1 expression is engaged by TRIM25. Indeed, TRIM25, by acting on the pro-caspase-1 CARD, could establish the reinforced interaction between pro-caspase-1 and ASC. Given that the RING domain, particularly C13 and C16, predicted to be critical for the catalytic activity of porcine TRIM25 was responsible for promoting the IL-1β response, TRIM25’s function to ubiquitinate its target protein(s) may be important. Although we could not observe pro-caspase-1 CARD ubiquitination (data not shown), whether it is subject to any post-translational modification as RIG-I CARD is ubiquitinated by TRIM25 (Gack et al., 2007) remains to be answered. If pro-caspase-1 is ubiquitinated and protected from degradation, there may exist more of pro-caspase-1 molecules that are available to interact with ASC. An additional possibility is that TRIM25 may form a complex with another cellular protein that associates with the pro-caspase-1 CARD. This complex may confer pro-caspase-1 better accessibility to ASC. In either case, pro-caspase-1 can be exposed to more chances of being recruited to be a part of the NLRP3 inflammasome complex, which leads to enhanced caspase-1 activity. Apart from these speculations, interestingly, an indirect participation of a TRIM protein in the regulation of NLRP3 inflammasome is reported. TRIM59 interacts with and ubiquitinates a cellular protein; degradation of this protein results in the NLRP3 inflammasome activation (Liang et al., 2020). For many TRIM proteins, their RING domain functions critically. Of note, the RING domain of TRIM25 in a fish species is responsible for the elevated transcription of pro-inflammatory cytokines including TNF-α and IL-6, but not IL-1β (Yang et al., 2016). In this context, whether TRIM25 has any effects on other cytokines and chemokines in pigs warrants further elucidation.

IAV NS1 protein is consisted of N-terminal RNA-binding domain (RBD) and C-terminal effector domain (ED); NS1 encoded by 2009 human pandemic IAV strains contains aa 1–73 as its RBD and aa 74–219 as the ED. The ED plays multiple roles by interacting with host proteins. For its association with TRIM25, contradicting patterns were observed for NS1 of a mouse-adapted IAV strain, A/Puerto Rico/8/1934/H1N1 (PR8); its ED alone fails to interact with TRIM25 (Gack et al., 2009) or can successfully bind TRIM25 (Koliopoulos et al., 2018). Previously, we showed that NS1 ED aa 100–219 of a 2009 pandemic strain, Hf09 could inhibit the NLRP3 inflammasome activation by acting on ASC (Park et al., 2018). Inhibitory roles of the NS1 C-terminal end on host inflammatory and antiviral responses are also reported with other IAV strains (Anastasina et al., 2015; Qian et al., 2017). In the current study, the NS1 C-terminal end of the Hf09 strain did not downregulate the expression of porcine or human TRIM25. Similar observations were reported for human TRIM25 using PR8 strain (Gack et al., 2009). It seems that TRIM25-mediated antiviral or inflammatory signaling upon IAV infection is regulated without remarkable changes in TRIM25 at the translational level. However, NS1 aa 100–219 of the Hf09 in our study could interact with TRIM25, leading to the status that pro-caspase-1 CARD-ASC interaction is disrupted. Altogether, our results revealed that regardless of E96/E97 residues, NS1 C-terminus can effectively suppress the NLRP3 inflammasome activity. Further efforts will be required to understand whether this behavior is strain-dependent since E96/E97 in the NS1 of PR8 strain are required to inhibit IL-1β response (Moriyama et al., 2016). Our and other studies conclude that TRIM25 is involved in the interplay between IL-1β and IFN responses as a previous report indicated that IL-1β production upon IAV infection is mediated by NLRP3 inflammasome as well as type I IFN signaling molecules (Pothlichet et al., 2013). Meanwhile, IAV NS1, especially the one encoded by 2009 pandemic strains, is equipped with strategies to counteract both responses, considering the IFN antagonism by NS1 of 2009 pandemic IAVs (Hale et al., 2010).

In this study, as summarized in the Figure 5, we report that TRIM25 positively regulates the NLRP3 inflammasome-mediated IL-1β production by increasing caspase-1 activity. While this upregulation was attributed to the enhanced interaction of pro-caspase-1 with ASC, it was counteracted by NS1 C-terminus of a 2009 pandemic IAV strain that interacts with TRIM25. This information extends our understanding toward the regulation of pro-inflammatory responses at the host-viral protein interaction level.

## Data Availability Statement

The original contributions presented in the study are included in the article/supplementary material, further inquiries can be directed to the corresponding author.

## Author Contributions

H-SP designed and performed the experiments, analyzed the data, and wrote the manuscript. YL, KP, and GL performed the experiments. YZ supervised the study, analyzed the data, and edited the manuscript. All authors contributed to the article and approved the submitted version.

## Funding

This research was supported by the Natural Sciences and Engineering Research Council of Canada (NSERC): RGPIN-2019-04578 funding to YZ. VIDO receives operational funding from the Government of Saskatchewan through Innovation Saskatchewan and the Ministry of Agriculture and from the Canada Foundation for Innovation through the Major Science Initiatives for its CL3 facility (InterVac).

## Conflict of Interest

The authors declare that the research was conducted in the absence of any commercial or financial relationships that could be construed as a potential conflict of interest.

## Publisher’s Note

All claims expressed in this article are solely those of the authors and do not necessarily represent those of their affiliated organizations, or those of the publisher, the editors and the reviewers. Any product that may be evaluated in this article, or claim that may be made by its manufacturer, is not guaranteed or endorsed by the publisher.
